# 2695. Lung Transplant Recipients With High Risk Cytomegalovirus Mismatch Donors Managed Using A Multimodality Regimen: A Five-Year Study

**DOI:** 10.1093/ofid/ofad500.2306

**Published:** 2023-11-27

**Authors:** Amit Banga, Rohan Kanade, Srinivas Bollineni, Vaidehi Kaza, Manish Mohanka, Irina Timofte, Adrian Lawrence, Fernando Torres

**Affiliations:** Stanford University, Stanford, California; University of Texas Southwestern Medical Center, Dallas, Texas; University of Texas Southwestern Medical Center, Dallas, Texas; University of Texas Southwestern Medical Center, Dallas, Texas; University of Texas Southwestern Medical Center, Dallas, Texas; University of Texas Southwestern Medical Center, Dallas, Texas; University of Texas Southwestern Medical Center, Dallas, Texas; University of Texas Southwestern Medical Center, Dallas, Texas

## Abstract

**Background:**

Lung transplant (LT) recipients with high risk cytomegalovirus (CMV) mismatched donors (recipient negative, donor positive or R-/D+) have been found to have worse early and late outcomes. In our institution, high risk CMV mismatch patients are managed in a protocolized manner consisting of proactive utilization of antiviral agents (ganciclovir/valganciclovir) and immune augmentation with CMV immune globulin.

**Figure:**
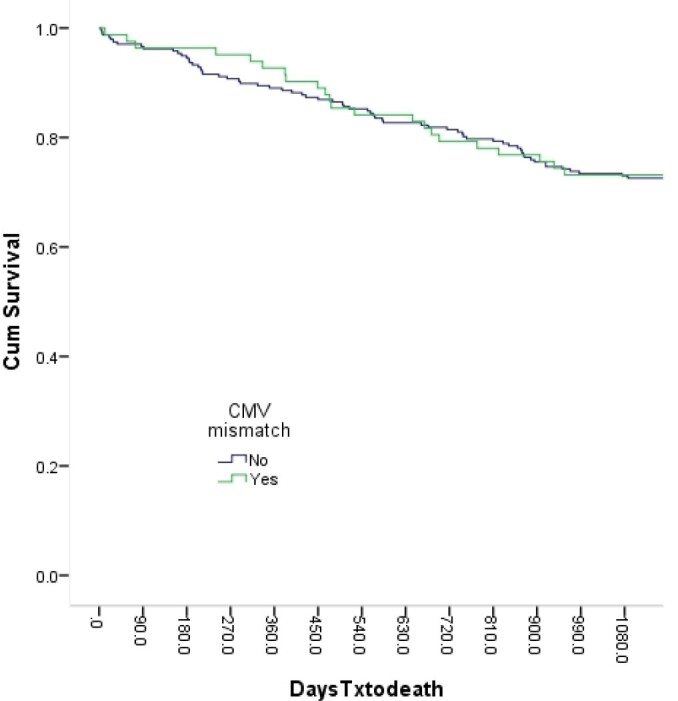

**Methods:**

We reviewed our institutional LT database. The study group consistent of all patients who underwent single or bilateral lung transplant between January 2012 to December 2016 (n=319). The CMV serostatus of both recipients and donors was reviewed and patients were classified into two groups: High risk CMV mismatch (R-/D+): n=82 (25.7%) and non-high risk CMV mismatch (n=237). We compared patient demographics, co-morbidities, pre and port-transplant variables among the two groups. Three-year survival was analyzed as the primary outcome variable. With three-year survival as the dependent variable, we analyzed the association of CMV status with survival using multivariate logistic regression analysis.

**Results:**

There was no difference in the baseline and post-transplant characteristics of LT recipients with and without CMV mismatch donors. Overall one-year and three-year survival was 89.96% and 72.7% respectively. Recipients transplanted with CMV mismatch status and managed with a proactive CMV prophylaxis protocol experienced similar one one-year (92.7% vs 89%, p=0.4) and three-year survival (73.2% vs 72.6%, p=1.0) as the non-CMV mismatch recipients.

After adjustment for demographics, comorbidities and post-transplant course, CMV mismatch was not associated with three-year survival. Post-LT development of AKI the only independent variable to be independently associated with three-year survival (adjusted OR: 2.1, 1.13-3.87; p=0.019). Kaplan Meier analysis (see Fig) showed very similar survival curves for recipients with and without CMV mismatched donors.

**Conclusion:**

Use of a proactive multimodality CMV prophylactic regimen consistent of antiviral agents (ganciclovir/valganciclovir) and immune augmentation with CMV immune globulin may improve outcomes among high risk CMV mismatch LT recipients.

**Disclosures:**

**All Authors**: No reported disclosures

